# Preclinical evaluation of lentiviral gene therapy for adenosine deaminase 2 deficiency (DADA2): engraftment efficiency and biodistribution in humanised NBSGW mice

**DOI:** 10.1038/s41434-025-00547-4

**Published:** 2025-06-24

**Authors:** Ying Hong, Alice Burleigh, Aiyin Liao, Jenny Yeung, Yixin Bian, Neil Sebire, Olumide Ogunbiyi, Ebun Omoyinmi, Adrian J. Thrasher, Emma Morris, Paul A. Brogan, Despina Eleftheriou

**Affiliations:** 1 Infection, Immunity, Inflammation Department, UCL Great Ormond Street Institute of Child Health, London, UK; 2 Department of Clinical Laboratory, Qilu Hospital of Shandong University Dezhou Hospital, Dezhou, China; 3 Department of Histopathology, Great Ormond Street Hospital for Children NHS Foundation Trust, London, UK; 4 UCL Institute of Immunity & Transplantation, London, UK

**Keywords:** Autoimmunity, Immunological disorders

## Abstract

Adenosine deaminase type 2 deficiency (DADA2) is caused by bi-allelic loss-of-function mutations in *ADA2*. While anti-TNF therapy is effective for the autoinflamatory and vasculitic components of the disease it does not correct marrow failure or immunodeficiency. Allogeneic stem cell transplantation (HSCT) offers a potential cure but is limited by challenges such as graft-versus-host-disease and donor availability. We previously demonstrated that lentiviral-mediated ADA2 gene therapy could restore ADA2 enzyme activity in patient-derived cells, correct macrophage inflammatory activation and reduce endothelial activation in vitro. Here, we evaluated the biodistribution and engraftment potential of lentivirally transduced healthy donor and patient-derived haematopoietic stem cells (HSC) in vivo using a humanised NBSGW mouse model. Transduced healthy HSC retained multilineage differentiation and engraftment capacity, without functional impairment. PCR analysis confirmed the absence of viral integration in non-haematopoietic organs, and histology showed no abnormal tissue changes, underscoring the safety and precision of this approach. In DADA2 patient-derived HSC, ADA2 transduction restored protein expression and enzyme activity, supporting improved cellular function and enhanced engraftment potential. These findings provide a strong foundation for advancing ADA2 gene therapy as a therapeutic strategy for DADA2, bringing it closer to clinical application.

## Introduction

Deficiency of adenosine deaminase type 2 (DADA2) is a rare genetic disorder that manifests as systemic autoinflammation, vasculitis, immune dysfunction, and haematologic abnormalities [1–3]. Treatment with anti-TNF is effective for the autoinflammatory and vasculitic components of the disease but does not correct marrow failure or immunodeficiency; and anti-drug antibodies can reduce efficacy over time [4–6]. Allogeneic haematopoietic stem cell transplantation (HSCT) may be curative, but graft versus host disease remains a significant concern and suitable donor matches are not always available [7–9]. Autologous gene therapy may offer a promising longer-term therapeutic solution by addressing the root cause of DADA2 through the restoration of ADA2 enzyme activity in haematopoietic stem cells (HSC). To explore this approach we previously conducted a proof-of-concept study to investigate whether lentiviral vector (LV)-mediated *ADA2* gene correction could rescue the immunophenotype of DADA2 in primary immune cells derived from patients and in cell line models [10]. We were able to show that lentiviral transduction led to: i) restoration of ADA2 protein expression and enzymatic activity; (ii) amelioration of M1 macrophage cytokine production, IFN-γ, and phosphorylated STAT1 expression in patient-derived macrophages; and (iii) amelioration of macrophage-mediated endothelial activation that drives the vasculitis of DADA2 [10]. We also successfully transduced human CD34 + HSC derived from a DADA2 patient with pure red cell aplasia and observed restoration of ADA2 expression and enzymatic activity in CD34 + HSC, alongside recovery of stem-cell proliferative and colony forming unit capacity [10].

Building on these preclinical findings, we aimed to further investigate the in vivo potential of the proposed gene therapy approach, a critical step toward evaluating its feasibility for clinical translation. However, the absence of an appropriate animal model that accurately represents human manifestations of DADA2 (due to the absence of a rodent orthologue for ADA2) necessitated alternative strategies to assess the engraftment efficiency, biodistribution, and functionality of gene-modified HSC. A widely accepted approach in the field involves using generic (non-disease specific) immunodeficient mouse models that lack mature T, B and NK cells and are permissive to human HSC grafts. Among these, the NBSGW mouse model (NOD.Cg-*Kit*^*W-41J*^*Tyr*^+^*Prkdc*^*scid*^*Il2rg*^*tm1Wjl*^/ThomJ; JAX strain #:026622) provides a valuable platform for in vivo testing of human HSC-based therapies [11–13]. Unlike other humanised mouse models like the NSG mice, NBSGW mice do not require irradiation providing additional advantages [11–13]. The NBSGW mouse model also supports superior engraftment, long-term survival, and effective multilineage differentiation, yielding high levels of human chimerism and higher frequencies of key HSC markers. In this study, we utilised these NBSGW mice to assess the biodistribution and engraftment of lentivirally transduced healthy donor HSC and HSC from a patient with DADA2. Our findings demonstrate that lentiviral transduction of healthy control HSC did not impair their engraftment potential or multilineage differentiation. Furthermore, lentiviral-mediated gene correction successfully restored ADA2 protein expression and enzyme activity associated with DADA2 in vivo, improving the engraftment potential and multilineage differentiation of patient-derived HSC. These results now provide strong proof of concept for the in vivo safety and efficacy of an autologous HSC gene therapy treatment approach for DADA2.

## Materials/patients and methods

### Patient samples

This study was approved by the Bloomsbury Ethics Committee (no. 08H071382). CD34 + HSC were obtained from the bone marrow of a 3-month-old severe DADA2 patient presenting with Pure Red-Cell Aplasia (PRCA) (genotype ADA2 p.G47W/p.G47W). Healthy donor HSC were obtained from leukapheresis provided from the Antony Nolan laboratories with consent (no. CGT125, 2 healthy donors: age 21 and 31 years old, one male, one female).

### Mice

NBSGW mice were obtained from the Jackson Laboratory. Procedures were approved by the UCL Biological Services Animal Welfare and Ethical Review Board and licensed by the Home Office under the UK Animals (Scientific Procedures) Act 1986, Amendment Regulations 2012 (ASPA). Transplanted mice were monitored daily during the first 2 weeks after transplantation and then weekly to record body weight. After 8- and 12-weeks post-transplantation, mouse peripheral blood from tail vein was stained with anti-human CD45-APC antibody (clone H130, Biolegend) to evaluate human CD45+ engraftment by flow cytometry. Mice showing weight loss of 20% or more, or signs of distress were humanely sacrificed following the NC3R recommendations.

### CD34+ HSC isolation, transduction, cell differentiation and colony forming unit (CFU) Assay

CD34 + HSC were isolated from mobilised peripheral blood for healthy donor cells (apheresis product, *n* = 2), and the bone marrow aspirate of a patient with DADA2 using magnetic separation following the manufacturer’s protocol (Miltenyi Biotec, UK). CD34^+^ cells were seeded in growth medium (SCGM + 1% human serum albumin, 20 ng/ml IL-3, 300 ng/ml stem cell factor [SCF], 300 ng/ml fms-like tyrosine kinase 3 ligand [FLT3L], and 100 ng/ml thrombopoietin [TPO]; Peprotech, UK) at a density of 1 × 10^6^ cells/ml and pre-stimulated overnight. CD34 + HSC transduction experiments were carried out by using a third-generation lentiviral vector on a pCCL backbone containing codon-optimized human *ADA2* cDNA driven by the elongation factor 1α short (EFS) promoter, internal ribosomal entry site (IRES), and enhanced green fluorescent protein (eGFP), or eGFP alone (EFS-ADA2-eGFP; EFS-eGFP). Vectors were produced by transient transfection of HEK293T cells as previously described [10, 14].

Cells were transduced with the lentiviral vector at MOI 30 in the presence of 4μg/ml of Protamine Sulphate and 1 mg/ml of LentiBOOST (GMP grade Kolliphor P338, BASF) for 16–18 h. After transduction, cells were frozen in CryoStor@CS10 (Stem Cell Technology, UK) for the transplantation experiment. Some cells were maintained in liquid culture in the growth medium for cell proliferation and viral copy number (VCN) assay, or grown as progenitors in semi-solid Methocult medium (STEMCELL) for 2 weeks as described previously [10]. Colony forming units (CFU) were enumerated by manual counting on day 14 post-plating, discriminating BFU-E (burst forming units-erythroid), CFU-GM (colony forming units granulocyte-macrophages) and CFU-GEMM (colony forming units granulocyte, monocyte, megakaryocyte). Total colonies counted were more than 50, using a density of 250 LV-GFP or LV-ADA2-GFP transduced cells in a total volume of 1.1 mL per well of a 6-well plate. Vector copy number (VCN) of LV-transduced cells was between 1.6 and 4.0.

### Western blotting

Cells were lysed in RIPA buffer (Thermo Fisher Scientific, Waltham, Mass) with 1% proteinase inhibitors (Roche Diagnostics). Lysates were boiled in the presence of 2 X Laemmli Buffer. Protein concentrations were measured using Pierce BCA Protein Assay (Thermo Fisher Scientific, UK). A total of 20 μg total protein was subjected to SDS-PAGE analysis and electrotransferred onto polyvinylidene difluoride membranes (Millipore, Temecula, Calif). Membranes were blocked with milk, probed with primary and secondary antibodies, and visualized with the enhanced chemiluminescence detection system (Amersham Pharmacia Biotech, Little Chalfont, United Kingdom). The following antibodies were used: ADA2 (ab154619, Abcam), ACTN (MAB 1501R; Merck Millipore, Burlington, Mass), Goat anti-Rabbit IgG, HRP (ThermoFisher) and Rabbit anti-mouse IgG, HRP (ThermoFisher).

### Transplantation into NBSGW Mice

Frozen untransduced/transduced CD34 + HSC were thawed, washed and resuspended in 200 μl of PBS (0.5 ×10^6^ per mouse) and injected via tail vein into NBSGW mice with a 29-gauge ×0.5 inch needle. A total of 21 mice (6 weeks old, females) were used in this study. Table 1 summarises the different experimental groups and conditions studied. Of note one mouse (transplanted with untransduced healthy donor HSC) was ultimately excluded from the study because of its mega-spleen and hyperactivity.

**Table 1:** Experimental groups of NBSGW mice and treatments received.

Groups	Number of NBSGW mice	VCN	Number of transplanted cells/mouse
Healthy donor CD34 + HSC untransduced	**8**	UT	0.5 × 10^6^ cells
Healthy donor LV-GFP	**3**	4.0	0.5 × 10^6^ cells
Healthy donor LV-ADA2-GFP	**8**	2.5– 2.8	0.5 × 10^6^ cells
DADA2 patient untransduced	**1**	UT	0.22 × 10^6^ cells
DADA2 patient LV-ADA2	**1**	1.6	0.22 × 10^6^ cells

*NBSGW* NOD.Cg-*Kit*^*W-41J*^*Tyr*^+^*Prkdc*^*scid*^*Il2rg*^*tm1Wjl*^/ThomJ, *HSC* haematopoietic stem cells, *LV* lentiviral, *HD* healthy donor, *GFP* Green fluorescent protein, *UT* untransduced, *ADA2* adenosine deaminase type 2, *DADA2* deficiency of adenosine deaminase type 2, *VCN* vector copy number.

### Engraftment studies and ADA2 Expression

A total of 50 μl blood sample was drawn into heparin-coated capillary tubes from the tail vein of all animals and processed for flow cytometric analysis. At 12-weeks post engraftment, serum, bone marrow, spleen, thymus, liver, lung, heart and brain were collected.

#### Flow cytometry

##### Detection of engrafted cells

Human cell engraftment was assessed by staining peripheral blood (PB), bone marrow (BM) and spleen cell suspension with the anti-CD45-APC antibody (clone H130, Biolegend). Cells were recorded on the CytoFLEX flow cytometer (Beckman Coulter, Buckinghamshire, UK), and data were analysed using FlowJo software (TreeStar, Ashland, OR, USA). The LOQ was set at >1% CD45+ cells.

##### Lineage distribution in NBSGW mice

Cell suspensions from BM, spleen and PB (~2 × 10^5^ cells) were co-stained in 50 µl MACS buffer with 0.5 µl of each of the following antibodies: CD45-APC, CD19-PE-Cy7 (Clone HIB19, Biolegend)), CD3-Pacific Blue (Clone OKT3, Biolegend), and CD33-PE (Clone VM53, Biolegend). Prior to acquisition, samples were stained with 2 µl of the viability dye DAPI. Samples were analysed on the CytoFLEX flow cytometer (Beckman Coulter, Buckinghamshire, UK), and data were analysed using FlowJo software (TreeStar, Ashland, OR, USA).

### ADA2 enzyme activity

A commercial assay kit was used to quantify serum ADA2 enzyme activity according to the instructions of the manufacturer (Diazyme, USA). The assay quantifies the adenosine-dependent generation of ammonia by coupling to the Glutamic Dehydrogenase (GDH)-catalysed reaction of NH3 with α-ketoglutarate in the presence of NADH. ADA2 activity is distinguished from total ADA with the use of the selective inhibitor of ADA1, 100 nM EHNA (erythro-9-Amino--hexyl--methyl-9H-purine-9-ethano hydrochloride). The kinetics of each reaction were analysed using an Optima Microplate Reader (BMG Labtech, UK).

### Histopathology

Formalin-fixed samples of thymus, spleen, liver, lung, liver and brain from 21 mice were processed by Great Ormond Street Hospital, Department of Histopathology. Slides from each of the samples were dewaxed in xylene, hydrated through a series of graded alcohols and rinsed in running tap water. The slides were then stained in Mayer’s haematoxylin before counter staining with aqueous eosin. Stained slides were then scanned on the Hamamatsu Nanozoomer 2.0 HT at 20x magnification to produce whole slide digital scans. Each of the 78 digital scans were evaluated by the study pathologist using the NDP.view2 viewing software. Each H&E section was thoroughly examined histologically, and lesions observed were recorded in an Excel spreadsheet, their severity graded (minimal, mild, moderate, or severe). Their distribution was also characterized (focal, multifocal, focally extensive or diffuse), as well as their localisation. Examination was performed in a blinded manner without knowledge of animal age, sex, strain, and/or treatment.

### Biodistribution studies

#### Genomic DNA extraction

gDNA from blood samples and/or haematopoietic organs (bone marrow, blood, spleen, thymus, kidney, lung, heart and brain) was extracted using the DNeasy Blood and Tissue Kit (QIAGEN) following the manufacturer’s protocol. DNA concentration for each sample was determined by Nanodrop (Thermo Scientific, USA).

#### VCN by qPCR

Average vector copy number/cell (VCN) was calculated as previously described [10]. The set of primers and probes used are listed below: psi copies/hAlb copies *2.

HIV-PSI Forward 5’ CAG GAC TCG GCT TGC TGA AG 3’, HIV-PSI Reverse 5’ TCC CCC GCT TAA TAC TGA CG 3’, HIV-PSI Probe 5’ FAM-CGC ACG GCA AGA GGC GAG G TAMRA-3’, H Albumin Forward 5’ GCT GCT ATC TCT TGT GGG CTG T 3’, H Albumin Reverse 5’ ACT CAT GGG AGC TGC TGG TTC 3’, H Albumin Probe 5’ VIC-CCT GTC ATG CCC ACA CAA ATC TCT CC-TAMRA 3’.

The limit of Detection (LOD) was set at >0.001 copies per µl, and the Limit of Quantification (LOQ) was fixed at <4 copies of human albumin per µl for vector copy number.

### Statistical analysis

All data were assembled and analysed using GraphPad Prism software version 10. Results were expressed as mean and standard error of the mean, or median and range. ANOVA and T-test were used for group comparisons. *P* values of less than 0.05 were considered significant.

## Results

### Transplantation of LV-ADA2 gene transduced healthy donor HSC in NBSGW mice has no adverse impact on body weight or tissue histology

**Fig. 1:**
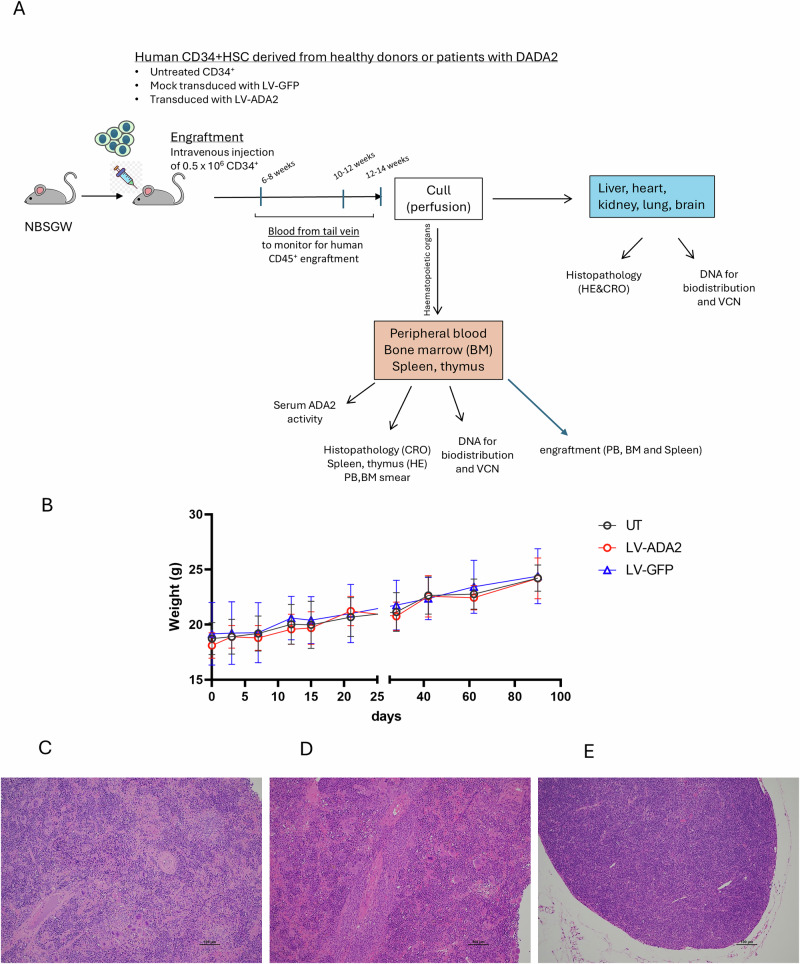
Body weight and histopathological analysis of NBSGW mice transplanted with LV-transduced CD34 + HSC derived from healthy donors. **A** Outline of the overall study design exploring the engraftment and biodistribution of LV-transduced HSC derived from healthy donors and patients with DADA2 in NBSGW mice. **B** Monitoring of body weight (g) over time in NBSGW mice transplanted with untransduced CD34 + HSC, LV-GFP-mock transduced CD34 + HSC, and LV-ADA2-transduced CD34 + HSC. Linear regression analysis demonstrated no difference in the rate of body weight change between any study group. **C**, **D** H&E ×100 and **E** ×40 original magnification images with scale. Histological sections showing examples of normal lymph node (**C**, **D**) and (**E**) spleen showing extramedullary haematopoiesis from 3 representative animals transplanted with untransduced CD34 + HSC, LV-GFP-mock transduced CD34 + HSC, and LV-ADA2-transduced CD34 + HSC groups. There were no significant histological abnormalities in any organ examined. All CD34 + HSC were derived from healthy donors. NBSGW NOD.Cg-*Kit*^*W-41J*^*Tyr*^+^*Prkdc*^*scid*^*Il2rg*^*tm1Wjl*^/ThomJ, HSC haematopoietic stem cells, VCN vector copy number, UT untransduced, LV lentiviral, GFP Green fluorescent protein, ADA2 adenosine deaminase type 2, DADA2, deficiency of adenosine deaminase type 2.

Initially, we examined the effects of transplantation with LV-transduced HSC derived from healthy donors on the general health and tissue histology of NBSGW mice. NBSGW mice were transplanted with untransduced CD34 + HSC, LV-GFP mock transduced CD34 + HSC, and LV-ADA2-transduced CD34 + HSC (Table 1 and Fig. 1A). The body weight of all groups was monitored for 90 days. We observed no differences in the rate of body weight change among the three study groups (Fig. 1B). Additionally, histological analysis of lymph node and spleen sections was performed on tissue from all study animals with no significant histological abnormalities detected; images from three representative animals are shown in Fig. 1C, E.

### LV-ADA2 gene transduced healthy donor HSC retain engraftment efficiency and multilineage differentiation post transplantation in NBSGW mice

**Fig. 2:**
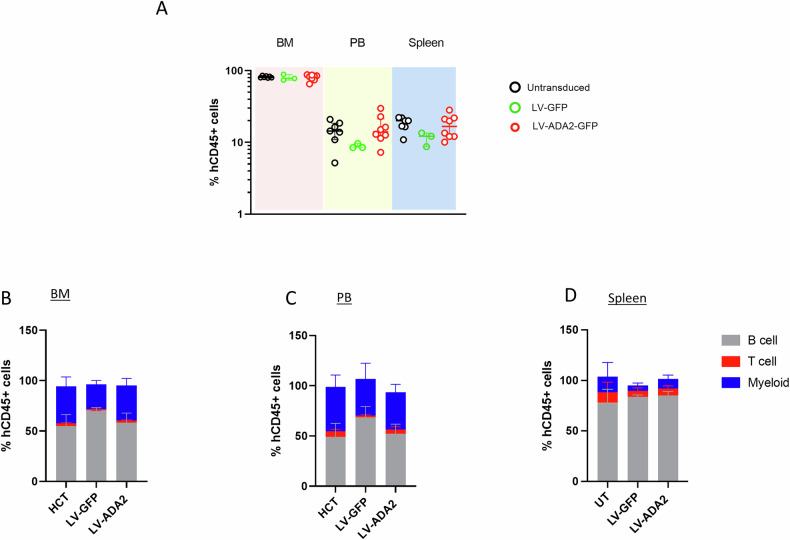
CD34 + HSC engraftment and multilineage differentiation in NBSGW mice transplanted with LV-transduced CD34 + HSC derived from healthy donors. Mice were injected with human CD34 + HSC cells derived from healthy donors, either untransduced (UT), LV-GFP-transduced, or LV-ADA2-transduced. **A** Engraftment of human cells was calculated based on the percentage of human CD45+ cells detected in different organs. LV-ADA2 gene correction did not significantly affect engraftment of human cells. **B**–**D** The percentage of the most representative subpopulations as detected in the bone marrow, peripheral blood and spleen are shown: T cells (CD3+), B cells (CD19+), and myeloid cells (CD33+). LV-ADA2 gene correction did not significantly affect multilineage differentiation of human cells. NBSGW NOD.Cg-*Kit*^*W-41J*^*Tyr*^+^*Prkdc*^*scid*^*Il2rg*^*tm1Wjl*^/ThomJ, HSC haematopoietic stem cells, LV lentiviral, GFP Green fluorescent protein, ADA2 adenosine deaminase type 2, BM bone marrow, PB peripheral blood, UT untransduced.

We next evaluated the engraftment potential and multilineage differentiation of LV-transduced HSC transplanted in NBSGW mice (Fig. 2). Engraftment of human cells was measured as the percentage of CD45+ cells detected in bone marrow, peripheral blood and spleen. We observed similar rates of CD45+ cells in the bone marrow across all groups: mice transplanted with untransduced healthy donor HSC (median 80.9, range 78.9–86%); mice transplanted with LV-GFP mock transduced HSC (median 79, range 74–88.9%); and mice treated with LV-ADA2 transduced HSC (median 82.2, range 67–87%), *p* = 0.7 (Fig. 2A). Similar rates of CD45+ cells were also observed in the peripheral blood and spleen of mice transplanted with untransduced HSC, LV-GFP mock transduced, and LV-ADA2 transduced HSC (Fig. 2A).

Furthermore, LV transduction did not significantly affect the multilineage differentiation of human HSC. The percentages of T cells (CD3+), B cells (CD19+), and myeloid cells (CD33+) detected in the bone marrow were similar in mice transplanted with untransduced HSC, LV-GFP mock transduced cells, and LV-ADA2 transduced HSC, (Fig. 2B and Supplementary Table 1). Similarly, no differences were observed between study groups in the peripheral blood or the spleen of treated mice (Fig. 2C, D and Supplementary Table 1). These results suggest that LV-ADA2 gene correction did not significantly impact the engraftment and differentiation of human cells post-transplant in NBSGW mice.

### Stable distribution of vector-bearing human cells in haematopoietic organs without accumulation in non-haematopoietic organs following LV-ADA2 gene transduction of healthy donor HSC

**Fig. 3:**
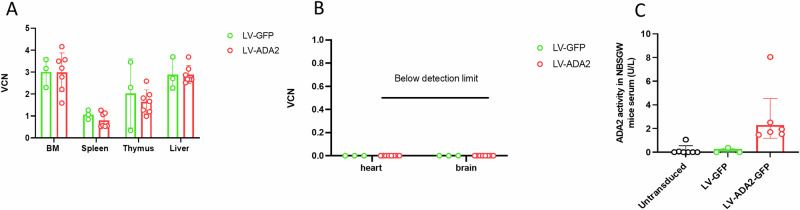
Biodistribution study in NBSGW mice transplanted with LV-transduced CD34 + HSC derived from healthy donors. **A** Viral copy number (VCN) per human cell was determined in haematopoietic organs (bone marrow, spleen, thymus, and liver), and **B** non-hematopoietic organs (lung, kidney, brain, and heart) by qPCR. There was no difference in the distribution of vector-bearing (human) cells in haematopoietic organs between study groups; the vector-bearing cells did not accumulate in non-haematopoietic organs including heart and brain. **C** ADA2 enzyme activity was measured in the serum of NBSGW mice transplanted with untransduced CD34 + HSC, LV-GFP-mock transduced CD34 + HSC and LV-ADA2-transduced CD34 + HSC. There was detectable ADA2 enzyme activity in the serum of mice receiving LV-ADA2-GFP transduced CD34 + HSC. All HSC were derived from healthy donors. NBSGW NOD.Cg-*Kit*^*W-41J*^*Tyr*^+^*Prkdc*^*scid*^*Il2rg*^*tm1Wjl*^/ThomJ, HSC haematopoietic stem cells, LV lentiviral, GFP Green fluorescent protein, ADA2 adenosine deaminase type 2, VCN vector copy number, BM bone marrow.

The VCN per human cell was determined in haematopoietic organs (bone marrow, spleen, thymus, and liver), and non-haematopoietic organs (brain, and heart) by qPCR (Fig. 3A, B). There was no difference in the distribution of vector-bearing (human) cells between study groups (mice transplanted with LV-GFP mock transduced HSC or LV-ADA2 transduced HSC) in bone marrow (median 3.1, range 2.3–3.6 versus median 3.4, range 1.6–4.2; *p* = 0.9), spleen (median 1, range 0.8–1.2 versus median 0.8, range 0.7–1.2; *p* = 0.3), thymus (median 2.2, range 0.8–3.4 versus median 1.8, range 0.9–2.5; *p* = 0.6), liver (median 2.7, range 2.3–3.7 versus median 2.6, range 2.5–3.8; *p* = 0.9).

Additionally, vector-bearing cells did not accumulate in non-haematopoietic organs including heart and brain, with undetectable VCN in these tissues for all study groups (Fig. 3B).

### LV-ADA2 gene transduction of healthy donor HSC yields detectable ADA2 enzyme activity in NBSGW mice serum

ADA2 enzyme activity was measured in the serum of NBSGW mice transplanted with untransduced CD34 + HSC, LV-GFP-mock transduced CD34 + HSC and LV-ADA2-transduced CD34 + HSC (Fig. 3C). Detectable ADA2 enzyme activity (median 2.867 U/L, range 1.47–8.05 U/L) was observed in the serum of mice transplanted with LV-ADA2 transduced cells; while there was no detectable enzyme activity in mice transplanted with LV-GFP mock transduced cells, or untransduced cells.

### LV-ADA2 gene transduction of DADA2 patient-derived HSC promotes engraftment and multilineage differentiation and restores ADA2 enzyme activity

**Fig. 4:**
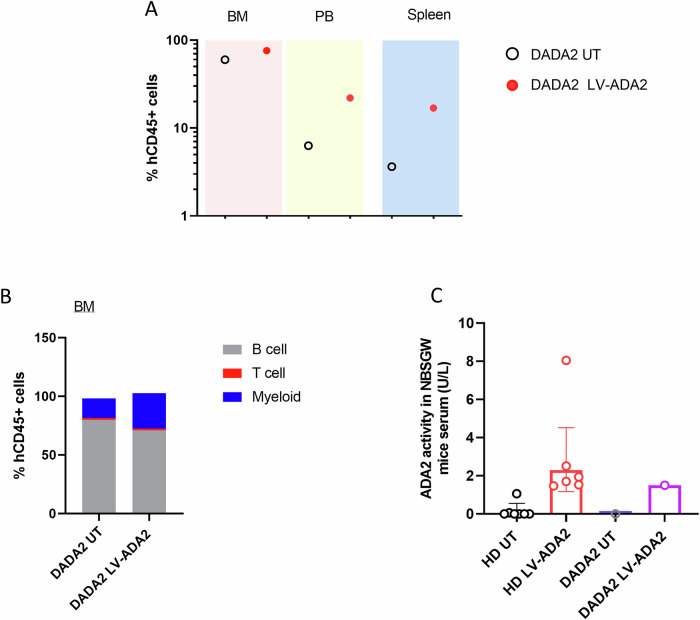
In vivo haematopoietic cell reconstitution in NBSGW mice transplanted with LV-ADA2 gene corrected CD34 + HSC derived from a patient with DADA2. **A** Engraftment, and **B** lineage distribution of human (hCD45+) cells in the bone marrow of NBSGW mice examined at 12 weeks following transplantation with untransduced CD34 + HSC or LV-ADA2-GFP gene corrected CD34 + HSC cells derived from a patient with DADA2. LV-ADA2-GFP gene correction promoted engraftment and multilineage distribution of CD34 + HSC derived from a patient with DADA2. **C** ADA2 enzyme activity in the serum of NBSGW mice transplanted with untransduced healthy donor cells (HD-UT); LV-ADA2-GFP transduced HSC (HD LV-ADA2); untransduced HSC from a patient with DADA2 (DADA2-UT); and LV-ADA2-GFP transduced HSC from a patient with DADA2 (DADA2-LV-ADA2). There was detectable ADA2 enzyme activity in the serum of mice that received LV-ADA2 transduced CD34 + HSC derived from a patient with DADA2, with no activity detected in the serum of mice receiving untransduced cells. NBSGW NOD.Cg-*Kit*^*W-41J*^*Tyr*^+^*Prkdc*^*scid*^*Il2rg*^*tm1Wjl*^/ThomJ, HSC haematopoietic stem cells, LV lentiviral, HD healthy donor, GFP Green fluorescent protein, UT untransduced, ADA2 adenosine deaminase type 2, DADA2 deficiency of adenosine deaminase type 2, BM bone marrow, PB peripheral blood.

The engraftment and lineage distribution of human (hCD45+) cells in the bone marrow of NBSGW mice were examined at 12 weeks following transplantation with either untransduced CD34 + HSC or LV-ADA2-gene transduced CD34 + HSC cells derived from a patient with DADA2 (Fig. 4A, B). LV-ADA2 transduction improved engraftment of CD34 + HSC and enhanced multilineage differentiation. Detectable ADA2 enzyme activity (1.5 U/L) was observed in the serum of mice transplanted with LV-ADA2 transduced HSC with no detectable activity in the serum of mice receiving untransduced cells (Fig. 4C).

## Discussion

Building upon our previous findings that lentiviral-mediated ADA2 gene correction restores enzyme activity and cellular function ex vivo, this study now provides compelling in vivo evidence supporting the safety and efficacy of a gene therapy approach for treating DADA2. Our experiments with transplanted NBSGW mice demonstrated that lentiviral-mediated ADA2 transduction of healthy donor HSC maintains their multilineage differentiation and engraftment potential. Importantly, we observed no adverse impact on HSC functionality following gene transfer. PCR analysis confirmed an absence of viral integration in non-haematopoietic organs, and histological assessments showed no unexpected tissue abnormalities, indicating that the lentiviral vector successfully targeted HSC without off-target effects. These findings underscore the safety and precision of our lentiviral gene therapy approach for DADA2, supporting its viability for future clinical applications.

For DADA2 patient-derived HSCs, transduction yielded encouraging outcomes. ADA2 protein expression and enzyme activity was effectively restored in these cells, demonstrating that lentiviral-mediated gene therapy can successfully reverse the protein/enzyme defect associated with DADA2. Notably, the modified HSC showed enhanced engraftment and differentiation potential, which suggests an intrinsic improvement in cellular function upon ADA2 gene restoration. In addition, for the first time, we detected ADA2 enzyme activity in the serum of mice that received LV-ADA2 transduced CD34 + HSC derived from a patient with DADA2, even if at lower levels compared to healthy human ADA2. No activity was detected in the serum of mice receiving untransduced cells. We acknowledge that while these data from the experiments with the patient-derived HSC suggest a positive effect, further studies with larger sample sizes would be needed to confirm statistical significance.

The NBSGW mouse model proved to be highly effective for evaluating the efficacy of our gene therapy approach. Compared to NSG mice, NBSGW mice, which combine the NOD SCID gamma immunodeficient strain with a B6 genetic background, have been shown in previous studies to support enhanced human HSC engraftment [11, 15]. This makes NSGBW mice particularly advantageous for studies requiring robust human immune cell reconstitution or gene editing applications. Additionally, their higher frequencies of essential HSC markers provide a reliable foundation for studying the safety and efficacy of gene therapy interventions [11, 13, 16, 17]. Given the thorough comparative evaluations conducted in previous studies between the commonly used NSG mice and the NSGBW mice, we chose not to replicate this comparison in our study. Instead, we harnessed the distinct advantages of NBSGW mice to directly evaluate the in vivo outcomes of our gene therapy approach.

Despite these promising results, certain limitations remain. The lack of an animal model that replicates the DADA2 pathology limits our ability to evaluate all potential clinical outcomes. Additionally, while NBSGW mice enable human cell engraftment, they do not replicate aspects of the human immune response that could influence gene therapy outcomes. Further studies will also need to assess long-term efficacy and stability of ADA2 expression in vivo. Lastly, for patients with DADA2, low numbers and poor quality of HSC-stemming from disease related bone marrow dysfunction, impaired differentiation, and limited ex vivo expansion pose significant challenges to achieving sufficient engraftment and therapeutic efficacy for gene therapy [18]. Addressing these issues may require strategies like optimizing HSC expansion protocols, using less toxic pre-conditioning regimens to allow for improved HSC engraftment and continuing anti-inflammatory therapies during transplantation to protect the engrafted HSC. We did not conduct second transplantation experiments to validate true stem cell functionality as the initial transplantation demonstrated strong and stable HSC engraftment [19]. Additionally, the limited number of patient-derived HSC available precluded both secondary transplants and further in vitro differentiation studies. While such experiments would have provided valuable functional insights into the correction of the DADA2 phenotype, the scarcity of available HSCs restricted our ability to perform these additional analyses. Lastly, comprehensive vector integration site analysis (ISA) will need to be performed during any future clinical trials as part of long-term safety monitoring and in accordance with regulatory guidelines.

In summary our data provide proof of concept of the in vivo safety and efficacy of a lentiviral-mediated gene therapy approach for the treatment of DADA2. Future steps will focus on scaling up these findings and optimizing and validating good manufacturing practice HSC protocols for DADA2 in preparation for first-in-human studies.

## Supplementary information

Supplement Table 1

## Data Availability

The authors confirm that the data supporting the findings of this study are available within the article [and/or] its supplementary materials.
