# Supplementary material for: Preclinical evaluation of lentiviral gene therapy for adenosine deaminase 2 deficiency (DADA2): engraftment efficiency and biodistribution in humanised NBSGW mice
Source: Gene Ther. 2025 Jun 24;32(6):664–71. doi: 10.1038/s41434-025-00547-4 (PMC12714571; doi:10.1038/s41434-025-00547-4)
Supplement: Supplementary file 1 — Supplement Table 1 [file 41434_2025_547_MOESM1_ESM.pdf]

Supplement Table 1: Percentage of human CD45+ cells at 12-Weeks post-transplant in hematopoietic compartment of NBSGW mice

Groups	Bone Marrow				Peripheral Blood				Spleen			
	%CD45	%CD3+ (T cell)	%CD19+ (B cell)	% CD33+ (myeloid)	%CD45	%CD3+ (T cell)	%CD19+ (B cell)	% CD33+ (myeloid)	%CD45	%CD3+ (T cell)	%CD19+ (B cell)	% CD33+ (myeloid)
**HD UT**	80.65 ± 4.37	2.76 ± 0.5	59.88 ± 8.24	46.84 ± 9.12	14.33 ± 5.18	5.27 ± 1.96	49.17 ± 13.33	44.14 ± 11.80	19.64 ± 5.84	11.96 ± 10.56	77.73 ± 13.57	17.95 ±15.01
**HD LV-GFP**	80.30 ± 7.71	1.24 ± 0.71	70.51 ± 2.55	35.13± 6.60	9.18 ± 3.40	6.51 ± 0.87	67.7 ± 7.42	36.2 ± 10.19	11.47 ± 2.47	5.89 ± 2.85	68.63 ± 24.1	11.55 ± 5.81
**HD LV-ADA2-GFP**	80.60 ±7.52	2.45 ± 0.44	58.15 ± 9.69	46.96 ± 11.32	14.80 ± 8.55	4.19 ± 2.74	52.35 ± 9.35	37.01 ± 7.98	17.36 ± 4.13	6.72 ± 2.59	85.10 ± 3.93	9.65 ± 3.91
**DADA2 UT**	60	1.56	80	16.8	6.29	11.0	63.2	29.6	3.64	4.65	73.2	5.91
**DADA2 LV-ADA2**	76	1.53	71	30.1	22.0	2.51	48.4	42.4	16.90	16.6	81.1	7.09
